# Acute hot joints on the medical take: tapping into the skills of our workforce

**DOI:** 10.1093/rap/rkad098

**Published:** 2023-12-09

**Authors:** Koushan Kouranloo, Jennifer Christie

**Affiliations:** School of Medicine, University of Liverpool, Liverpool, UK; Liverpool University Hospitals NHS Foundation Trust, Liverpool, UK; School of Medicine, University of Liverpool, Liverpool, UK; Liverpool University Hospitals NHS Foundation Trust, Liverpool, UK

Key message

- Educating the wider MDT is an efficient updated IMT curriculum adaptation.

Dear Editor, Acute oligoarthritis is a common presentation to secondary care, with septic arthritis the most serious cause. The inpatient mortality from septic arthritis is 7–15%, despite antibiotics use [1]. Bacterial arthritis in the UK has an incidence of 1 in 49 000/100 000 person-years [2]. Arthrocentesis remains the gold standard in differentiating septic from non-septic causes. Prompt diagnosis of acute oligoarthritis remains fundamental in avoiding unnecessary antibiotics and reducing hospital admissions [3]. Acute oligoarthritis is managed by medical teams in many National Health Service (NHS) hospitals. Additionally, more common causes of acute hot joints, e.g. gout and pseudogout, often arise in elderly and multimorbid patients, in whom an important differential is septic arthritis, highlighting the need for urgent arthrocentesis.

Arthrocentesis was incorporated within the compulsory curriculum for Core Medial Trainees (CMT) in 2009 in the UK. Since implementation of Internal Medicine Training (IMT) and its new curriculum, this requirement has been removed for all IMT trainees. The rationale for this was delivering a more holistic training focused on ‘a small number of high-level learning outcomes rather than a large number of granular competencies’ [3]. Therefore, trainees often progress to the IMT3 level and above without having performed or observed arthrocentesis. Yet they are expected to run the acute unselected medical take with remote consultant supervision to meet the requirement at their Annual Review of Competency Progression [4].

Meanwhile, in comparison to the UK’s IMT with its North American Internal Medicine (IM) counterparts, arthrocentesis remains a core competency for IM certification in the USA and Canada [5].

The importance of managing patients within a multidisciplinary team (MDT) is well known to physicians. Since the introduction of Allied Health Professional (AHP) roles such as Advanced Nurse Practitioners (ANP) in 1998 and Physician Associates (PA) in 2003, they have been integrated as valuable members of the acute medical team, representing continuity of care for their departments [6, 7]. This is in stark contrast to trainee doctors who rotate through different specialties, hospitals and sometimes regions during their time until consultancy. Therefore, we argue that providing education to AHPs, in particular to those helping with the acute unselected take, could prove beneficial for patient care.

We developed a practical teaching program on managing acute oligoarthritis in our local tertiary rheumatology centre. The session lasted for 2 h. It was delivered with the help of one of the consultant rheumatologists with extended experience in managing hot joints as well as the personnel at the local postgraduate education centre, who provided appropriate hot joint models and kits to enable practical delivery. A model of the knee was used to simulate a hot joint presentation, as the most common site of presentation of acute oligoarthritis. The session was advertised to all AHPs as part of the acute unselected take.

We gathered pre- and post-session data on the participants’ confidence on arthrocentesis, ranging from ‘not at all confident’, ‘a little confident’, ‘quite confident’ and ‘somewhat confident’. We also gathered information on previous teaching or exposure in managing acute oligoarthritis, with the opportunity to elaborate on their experience as needed (Fig. 1).

**Figure 1.**
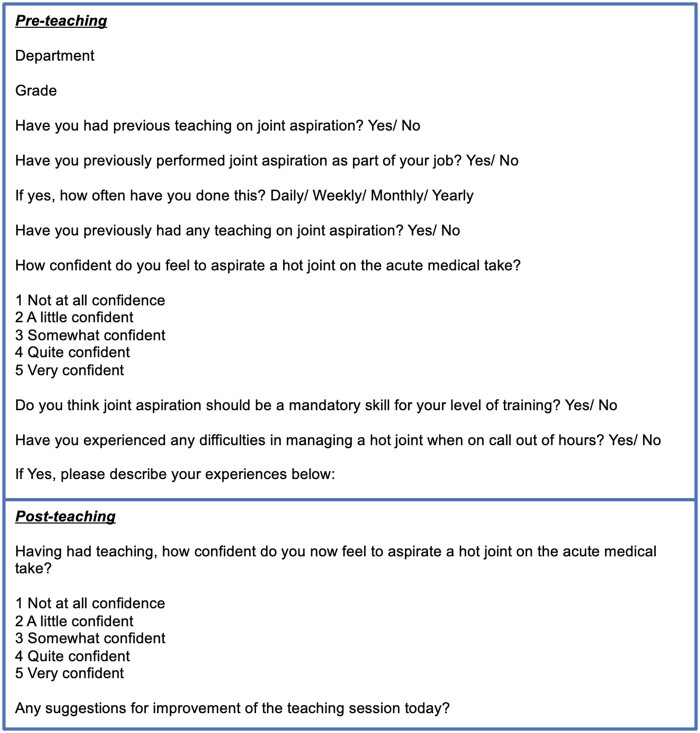
Pre- and post-teaching session surveys

Post-session, there was a 50% increase in participants’ confidence in managing an acute knee arthrocentesis, with five people (out of eight) identifying themselves as ‘quite confident’. There was only one person who continued to identify as ‘a little confident’ post-session. The remaining two identified as ‘somewhat confident’. No one identified as ‘not at all confident’ on their post-session survey responses (Fig. 2).

**Figure 2.**
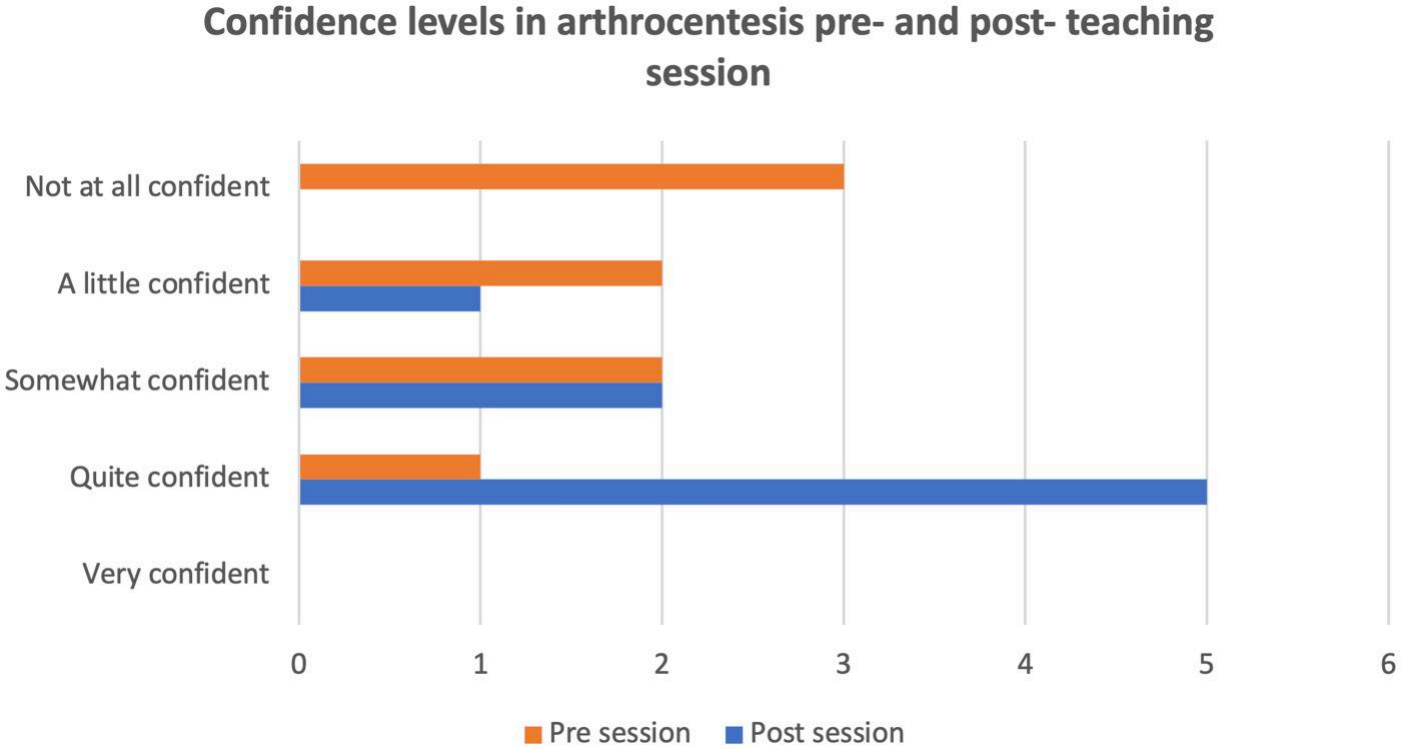
Confidence levels in arthrocentesis

Most of our attendees (87%) thought that arthrocentesis of the hot large joint should be a compulsory skill for their level of training.

With the recent changes in the shape of training leading to omission of certain skills such as arthrocentesis for IMT doctors, coupled with the ageing multimorbid UK population, we propose the need to educate AHPs who frequently deal with acute oligoarthritis as part of the unselected take. While the optimal solution would be to provide junior doctors with the necessary experience and skills in arthrocentesis, clinical pressures and the devaluation of the procedure in the IMT curriculum makes it vital to train AHPs, for both the benefit of the team and patients. In our centre, due to positive feedback and tangible benefits to the acute medical team, this practical skill session has been successfully delivered three times a year in conjunction with junior doctors’ changeover periods. This continues to add additional opportunities for AHPs, where they are encouraged to keep a portfolio of directly observed procedures and, once independent, are empowered to teach this skill to others. We have demonstrated an effective practical teaching program to accommodate for potential gaps as the result of the IMT curriculum change. We believe that this session can be adapted by other trusts in which management of acute oligoarthritis falls within the realms of medical teams.

## Data Availability

All data are available upon reasonable request.
